# Lifestyle management to prevent atherosclerotic cardiovascular disease: evidence and challenges

**DOI:** 10.1007/s12471-021-01642-y

**Published:** 2021-11-11

**Authors:** T. J. van Trier, N. Mohammadnia, M. Snaterse, R. J. G. Peters, H. T. Jørstad, W. A. Bax

**Affiliations:** 1 Department of Cardiology, Amsterdam University Medical Centre, University of Amsterdam, Amsterdam, The Netherlands; 2 Department of Internal Medicine, Northwest Clinics, Alkmaar, The Netherlands; 3 Vascular Research Alkmaar, Alkmaar, The Netherlands; 4 Department of Cardiology, Northwest Clinics, Alkmaar, The Netherlands; 5 Amsterdam School of Health Professions, University of Applied Sciences, Amsterdam, The Netherlands

**Keywords:** Lifestyle, Prevention, Risk management

## Abstract

Lifestyle management is the cornerstone of both primary and secondary prevention of atherosclerotic cardiovascular disease (ASCVD) and the importance of lifestyle management is emphasised by all major guidelines. Despite this, actual implementation of lifestyle management is poor. Lifestyle modification includes smoking cessation, weight loss, dietary change, increasing physical inactivity, and stress management. This review summarises evidence-based opportunities and challenges for healthcare professionals to promote healthy lifestyles at an individual level for the prevention of ASCVD.

## Introduction

Diet-related risks, tobacco and alcohol use, and physical inactivity are responsible for more than 40% of disability-adjusted life years in high-income countries [1]. Approximately 90% of cardiovascular risk is attributable to potentially modifiable risk factors; lifestyle-related factors account for more than half of this risk [2]. At the population level, unhealthy lifestyles constitute a problem of contemporary society, which ideally requires concerted, multi-level prevention efforts. Yet, at the individual level, the responsibility of healthcare professionals is undeniable. Hence, all major clinical guidelines on primary or secondary prevention of atherosclerotic cardiovascular disease (ASCVD) emphasise the importance of lifestyle modification in clinical practice [3]. Despite this emphasis, disappointing results prevail in implementation and adherence to lifestyle interventions in daily practice, both in primary [4] and secondary [5] prevention. This review summarises evidence-based opportunities and challenges for healthcare professionals to promote smoking cessation, weight loss, dietary change, physical activity, and stress management in patients for prevention of ASCVD.

## Risk assessment

The 2016 ESC prevention guidelines recommend formal risk assessment to take place prior to initiating specific lifestyle interventions. Consequently, with higher baseline cardiovascular risk levels, the absolute cardiovascular risk reduction achieved by successful lifestyle modification increases. Cardiovascular risk can be assessed using tools such as Systemic COronary Risk Estimation (SCORE) for individuals without prior ASCVD or SMART/SMART-REACH (https://u-prevent.com) for patients with known ASCVD. While guidelines do not provide a universally applicable threshold for the ASCVD risk requiring a certain ‘dosage’ of lifestyle interventions, the intensity of lifestyle interventions should generally increase with increasing ASCVD risk.

Lifestyle interventions should be tailored to an individual patient’s needs but may also be directed at groups instead of individuals. This emphasises the importance of identifying relevant (sub)groups (e.g. individuals < 50 years of age, in low socioeconomic positions, the elderly, female-specific conditions, ethnic minorities) that may particularly benefit from a specific lifestyle intervention. A single risk factor in a homogeneous patient population may be amenable to a variety of successful interventions, yet with sometimes minimal effects in a more diverse population. For example, the randomised RESPONSE‑2 trial demonstrated a complex interplay between successful weight loss and patient characteristics such as partner status, active partner participation, and age [6]. However, clinical trials investigating lifestyle modification in ASCVD patients often ignore differences in efficacy between specific groups. Consequently, the 2016 ESC Guidelines on CVD Prevention in Clinical Practice marked this issue as an important gap in current evidence, stating: ‘There is limited evidence to determine which interventions are most effective in specific groups (e.g. young–old, male–female, high vs low socio-economic status)’ [3].

In summary, both the estimated ASCVD risk and the subgroup-related probability of successful lifestyle modification may assist the healthcare provider in selecting the most appropriate lifestyle interventions, and may also prevent futile or even deleterious attempts to intervene in individuals with a low probability of success.

## Smoking cessation

Smoking is a leading cause of death worldwide and the most important modifiable risk factor for ASCVD. Smoking cessation either before or after acute myocardial infarction is associated with improved survival [hazard ratio (HR) 0.63, 95% confidence interval (CI) 0.48–0.82], and each reduction of five cigarettes daily after acute myocardial infarction is associated with an 18% decline in mortality risk over a median follow-up period of 13 years (*p* < 0.001) [7]. Compared with cholesterol-lowering and hypertension management, smoking cessation is by far the most cost-effective strategy [3]. Consequently, smoking cessation in ASCVD patients is arguably the most important preventive measure in cardiovascular risk management [8].

The EUROASPIRE surveys have shown that the majority of contemporary acute coronary syndrome (ACS) patients receive personal advice and counselling to stop smoking [9]. Yet, cessation rates post-ACS have remained unchanged at around 50% since 1999 [5, 9]. Interestingly, patients who quit immediately after their acute coronary event require the least smoking cessation support and constitute the majority of successful quitters (i.e. former smokers who remain abstinent through 1 year of follow-up) [10]. Smokers who have successfully quit do not require additional support to avoid relapsing [11]. A substantial potential to further reduce cardiovascular risk therefore lies in strategies focussed on persistent smokers and those who relapse.

With relatively little effort, healthcare professionals can facilitate small yet meaningful increases in cessation rates (Tab. 1). Even the simple advice to stop smoking, without any added assistance, has been shown to increase absolute cessation rates by 2–3% [12]. Furthermore, a meta-analysis of eight trials investigating smoking cessation rates showed that nurse-coordinated care improves smoking cessation rates by 25% [pooled relative risk (RR) 1.25, 95% CI 1.08–1.43] [13]. National and international guidelines recommend the ‘Five A’s’ framework, which includes: (1) asking every patient about tobacco use, (2) advising all tobacco users to quit, (3) assessing the willingness of all tobacco users to make an attempt to quit, (4) assisting tobacco users with their attempt to quit, and (5) arranging follow-up [3].

**Table 1:** Evidence-based interventions to enhance smoking cessation rates. NRT nicotine replacement therapy

Population	All smokers aged ≥ 18 years (both primary and secondary prevention)
Recommendation	Provide behavioural interventions for cessation in combination with pharmacotherapy: NRT, varenicline and bupropion (or a combination thereof)
Assessment	The 5 A’s framework: (1) ask about tobacco use, (2) advise to stop, (3) assess the willingness to quit, (4) assist the attempt to quit, (5) arrange follow-up
Behavioural counselling interventions	– More extensive behavioural counselling has not been shown to be more effective than brief interventions. – Both individual and group behavioural interventions are effective in helping smokers quit. – Nurse-coordinated care could enhance cessation rate. Support from the individual’s partner and family is important
Self-help material	Generic self-help material has no more effect than brief stop-smoking advice
Pharmacotherapy interventions	All forms of NRT are effective. The antidepressant bupropion has a similar effect to NRT. Varenicline at the standard dose—or low dose to reduce side effects—is more effective than NRT or bupropion. Combining two types of NRT is more effective than using a single type, and as effective as using varenicline
Other recommendations	Most effective is a combination of brief behavioural counselling and drug therapy, especially in patients recruited in healthcare settings, using the Five A’s framework

In addition to behavioural interventions, nicotine replacement therapy (NRT), varenicline or bupropion are evidence-based drug interventions. When compared to pharmacologically unassisted controls, the relative rate of successful long-term smoking cessation is enhanced by 50–60% with all forms of NRT [14] or bupropion [15] and even two- to threefold when using varenicline or combining two types of NRT [16]. However, the absolute effects of medication vary considerably and may be larger when the chance of success is enhanced by intensive behavioural support [14]. This combination of behavioural intervention with pharmacological support has been shown to be the most effective in achieving smoking cessation (RR 1.83, 95% CI 1.68‑1.98), with greater effect in patients recruited in healthcare settings [17]. No differences were detected between subgroups defined by motivation to quit, treatment provider, number or duration of support sessions or take-up of treatment. Despite high-quality evidence for the effect of combined therapy, actual implementation is limited, with reported implementation rates in 14% of smokers attempting to quit [18].

Evidence on the efficacy of electronic cigarettes to achieve cessation of smoking conventional cigarettes shows limited success rates and suggests potential for cardiovascular harm, for example through mechanisms that increase the risk of thrombosis [19]. There are no reliable data indicating that acupuncture, acupressure, laser therapy, hypnotherapy or electrostimulation are effective for smoking cessation [3].

In summary, the benefits of smoking cessation have been extensively demonstrated, and higher absolute cessation rates could be achieved in particular with a combination of behavioural and pharmacological support. The fact that a life-threatening event is insufficient for half of the smokers to quit reflects the complexity of smoking behaviour and the need for a personalised approach. Yet, every health care professional must always address the patient’s smoking behaviour, at least during every hospitalisation or revascularisation procedure.

## Weight loss interventions

Both overweight (BMI 25–30 kg/m^2^) and obesity (BMI > 30 kg/m^2^) are associated with an increased risk of metabolic risk factors (blood pressure, blood lipids, type 2 diabetes), ASCVD-related and all-cause mortality. Because all-cause mortality has been shown to be lowest within the BMI range of 20–25 kg/m^2^, this is the recommended target in ASCVD prevention in individuals < 60 years of age; at older ages the optimal BMI level is less clear [20].

Obesity trends are estimated to offset the positive effects of declining smoking rates [21]. While dietary habits in the Netherlands have generally improved and 15% of adults are on a diet [22], overweight has increased over time. Thus, 14% of the population older than 20 years in the Netherlands meet criteria for obesity, approximately 2.5 times more than during the 1980s [23].

Creating a caloric deficit remains the cornerstone of weight-loss strategies. Exercise can be helpful in creating a higher caloric demand, whereas diets for weight loss focus on caloric intake. The RESPONSE‑2 and Look AHEAD trials demonstrated that weight loss can be obtained with dietary interventions in secondary prevention, in the short and long term, respectively. In the RESPONSE‑2 trial, 181 overweight or obese coronary patients were randomised to a community-based commercial weight loss intervention (Weight Watchers); 27% had ≥ 5% weight reduction after 1 year versus 14% of controls with usual care (*p* *<* 0.001) [6]. Whether the effect remains at 3‑year follow-up is currently being analysed. In the Look AHEAD trial, 5145 overweight or obese patients with type 2 diabetes were randomised to participate in a lifestyle intervention aimed at weight loss by decreasing caloric intake and increasing physical activity, or to the control group [24]. After a median follow-up of no less than 9.6 years, greater weight loss was demonstrated in the intervention group as compared with the control group (8.6% vs 0.7% after 1 year; 6.0% vs 3.5% at the end of the study), together with greater reductions in glycated haemoglobin. The overall study outcome as regards reduction in cardiovascular events was neutral. A meta-analysis of randomised controlled trials aimed at weight reduction through dietary interventions with or without exercise programmes found that weight-loss interventions resulted in a decrease in premature mortality, but found no clear association with reduction in cardiovascular mortality or cardiovascular events [25]. As such, challenges in implementation and long-term adherence to dietary interventions remain.

Since diet and exercise are often unsuccessful in improving long-term outcomes, medical therapy or surgery should be considered, especially in patients with type 2 diabetes. When used in combination with lifestyle interventions, the use of liraglutide, orlistat and bupropion/naltrexone contributes to additional weight loss of approximately 3–9% relative to placebo after 1 year [26], but data are lacking on effects on ASCVD events as well as on long-term weight loss. Surgical interventions to reduce overweight are currently regarded as a last resort when the lifestyle strategies have failed. It is debatable whether such interventions may be regarded as ‘lifestyle modification’, and short- and long-term complications should be taken into account. However, bariatric surgery is an effective and sustainable treatment option for obesity. Bariatric surgery results in long-term weight loss, improvements in type 2 diabetes outcomes [27], and a reduction in cardiovascular events and deaths [28].

In conclusion, despite a clear association between overweight and ASCVD, there is surprisingly little evidence regarding sustainable implementation of effective strategies to achieve long-term healthy weight. Moreover, there is a lack of clear evidence that dietary interventions exclusively aiming at weight loss reduce the burden of ASCVD. The effects of pharmacological weight-loss strategies on ASCVD have yet to be proven, and while the efficacy and safety of bariatric surgery are well documented, the invasive nature of this procedure requires careful selection and shared decision-making with patients.

## Dietary change

Most diets, associated with cardioprotective effects, are low in saturated and trans fatty acids and high in polyunsaturated fatty acids (which mainly affect lipoprotein levels), low in sugar and alcohol, low in sodium, high in potassium (which mainly affect blood pressure), and enriched with vitamins and fibres (Tab. 2). Of note is that most evidence on the relation between ASCVD and nutrition is based on observational studies and often biased by confounding factors; evidence from randomised controlled trials investigating the impact on clinical endpoints is relatively scarce.

**Table 2:** Food with specific nutrients, targets, effects

Food groups	Specific nutrients	Targets	Effects of food	Diets
*Recommended to avoid*				
Margarine Bakery products Soft drinks	Trans unsaturated fatty acids Sugar	< 1% of total energy intake < 10% of total energy intake	Increase LDL-c/TC Lower HDL‑c Increase triglycerides	DASH
*Recommended to minimise*				
Red meat Dairy products	Saturated fatty acids	< 10% of total energy intake	Increase LDL‑c Increase HDL‑c	Mediterranean, DASH
Alcoholic beverages	Alcohol	≤ 2 men, ≤ 1 women units per day	Increase blood pressure	Mediterranean (moderate)
Processed food	Sodium	< 2 g/day	Increase blood pressure	DASH
*Recommended to increase*				
Fish (oil), plant oil, nuts	Unsaturated fatty acids	(Oily) fish 1–2 times/week; 30 g unsalted nuts/day	Lower LDL‑c Lower HDL‑c	Mediterranean
Fruits and vegetables	Potassium Vitamins A,C (antioxidant) B_6_, B_12_, folic acid, D	3.5–4.7 mg/day > 200 g fruit/day (2–3 servings); > 200 g vegetables/day	Lower blood pressure Lower oxidative stress Lower homocysteine	Mediterranean, DASH
Wholegrain products	Fibres	30–45 g/day	Lower TC Lower LDL‑c Reduce postprandial glucose response	Mediterranean

*LDL‑c* low-density lipoprotein-cholesterol, *TC* total cholesterol, *HDL‑c* high-density lipoprotein cholesterol, *DASH* Dietary Approaches to Stop Hypertension

### Shifting targets

Current dietary recommendations are continually being debated and revised, such as those for salt and alcohol. For example, the current World Health Organisation guidelines recommend a low daily sodium intake (< 2.0 g) and a high daily potassium intake (> 3.5 g) based on meta-analyses that found strong evidence supporting a linear dose–response relationship between reduction of sodium intake and blood pressure [29] and a study showing that a switch from regular (sodium-containing) salt to potassium-enriched salt resulted in a significant reduction of cardiovascular events and death in a large high-risk Chinese population [30]. By contrast, results from a recent prospective cohort study among 103,570 participants indicate that simultaneously reaching the dietary targets for both sodium and potassium is extremely uncommon, while the combination of moderate sodium intake (3–5 g/day) and high potassium intake is associated with the lowest risk of mortality and major cardiovascular events [31].

Another ongoing debate concerns the beneficial effect of moderate alcohol consumption compared with non-drinkers, as suggested by epidemiological studies [32], although confounding and reverse causality could not be excluded. A Mendelian randomisation study, analysing 59 epidemiological studies, reported that the lowest risk for ASCVD outcomes is in abstainers and any amount of alcohol is associated with elevated blood pressure and BMI [33]. An additional meta-analysis, comprising 600,000 individuals, found such harmful effects of drinking on cardiovascular outcome that the authors argued that difficulties determining the optimal target would have to result in lowering the recommended dose of alcohol consumption in existing guidelines [34]. For several subgroups (all but men aged between 41 and 65) there is evidence that light to moderate alcohol consumption has no protective effect on ASCVD [35], suggesting that the optimal amount of alcohol for the prevention of ASCVD may in fact be closer to ‘none’ than the ‘moderate’ amount recommended by conventional guidelines.

### Dietary combination interventions

The combined impact of several favourable dietary habits is discussed on the basis of three extensively studied dietary interventions aiming to prevent cardiovascular disease: the Mediterranean diet, the Dietary Approaches to Stop Hypertension (DASH) diet, and time-restricted eating. The Mediterranean diet comprises a high intake of fruits, vegetables, wholegrain products, fish, and unsaturated fatty acids (especially olive oil); moderate consumption of alcohol (mostly wine, preferably consumed with meals); and low consumption of (red) meat, dairy products, and saturated fatty acids. The Mediterranean diet results in consumption of significantly less saturated fat, cholesterol, and linoleic acid, but more oleic and alpha-linolenic acids, and is associated with a significant reduction of cardiac death, non-fatal myocardial infarction, and overall mortality up to 4 years after the first infarction [36]. Major traditional risk factors, such as blood cholesterol and blood pressure, were independently associated with recurrence of ASCVD events, indicating that the Mediterranean dietary pattern did not affect the usual relationships between major risk factors and ASCVD events [37]. Beneficial effects on major cardiovascular events of an energy-unrestricted Mediterranean diet, supplemented with olive oil or mixed nuts, were confirmed in the primary prevention PREDIMED study in patients at high risk for ASCVD events (29% lower risk of ASCVD over a 5-year period) [38]. Currently, the PREDIMED-Plus trial is investigating the effects of both an intensive weight-loss lifestyle intervention, based on an energy-restricted Mediterranean diet, stimulation of physical activity and behavioural support, as compared with a control group receiving educational sessions on Mediterranean diet only. Results are expected after 2022 [39].

The DASH diet, rich in fruits, vegetables, and low-fat dairy foods, and with reduced saturated, trans and total fat, was originally found to be able to substantially lower blood pressure [40], even more by additional reduction of sodium intake [41]. Although the original DASH publication [40] calculated a potential reduction of incident coronary heart disease by approximately 15% and of stroke by approximately 27%, no randomised controlled trials on the DASH diet and major adverse cardiovascular events have been published. Still, the DASH diet has been included in numerous guidelines in cardiovascular medicine, probably as a consequence of favourable adherence to the diet and an association with a lower risk of ASCVD, observed among middle-aged women [42]. On the other hand, in a recent large prospective study, adherence to a DASH-style diet was significantly associated only with all-cause mortality but not cardiovascular mortality [43].

Time-restricted eating (also referred to as intermittent fasting) has been suggested as a potential strategy for prevention of multiple diseases. Intermittent fasting potentially results in minimisation of anabolic processes and induces evolutionarily conserved cellular responses that improve glucose regulation, increase stress resistance, and suppress inflammation in favour of repair and elimination of damaged molecules [44]. Whether the effects of time-restricted eating are due to a change in metabolism or to weight loss remains uncertain. Intermittent energy and carbohydrate restriction has been shown to result in improved insulin sensitivity and body fat reduction as compared with daily energy restriction [45]. However, these results have not been replicated. Of note is that a recent randomised trial reported that time-restricted eating was not associated with greater overall weight loss but did result in greater decreases in lean body mass (fat-free mass) as compared with three meals per day [46]. Further studies are needed to determine whether promising pre-clinical and animal data translate into true improvement of cardiovascular health, or whether the concept of time-restricted eating reflects a romantic view of pre-evolutionary challenges of *Homo sapiens* food-gathering as opposed to the contemporary affluent supply of food, a situation which is undeniably related to adverse metabolic and cardiovascular outcomes.

In conclusion, dietary habits influence the risk of ASCVD, and a healthy diet is recommended to all individuals for ASCVD prevention. General characteristics of healthy food components that have protective effects may be well known, but challenges remain in identifying the detailed specifics of a healthy diet and developing effective strategies to facilitate adherence.

## Promoting physical activity and exercise

Cardiovascular risk is increased by sedentary behaviour and reduced by engaging in physical activity and sports. Healthcare professionals tend to emphasise the health benefits of exercise rather than the harmful effects of sedentary behaviour [47]. Indeed, regular physical activity prevents ischaemic heart disease [48], impacts favourably on numerous risk factors [49, 50], and has a dose-response impact on ASCVD events, all-cause and cardiovascular mortality in the general population [51] and in cardiac patients [52]. The association between physical activity and ASCVD risk appears to be stronger in women than in men [51]. In overweight and obese patients, the influence of physical activity and fitness on all-cause mortality appears to be greater than that of a high BMI. The strong evidence on the effects of sedentary behaviour calls for an active approach by physicians in warning against the harm of inactivity—comparable to the efforts against smoking [47].

Physical inactivity (also called sedentary behaviour)—defined as < 150 min of moderate-intensity or < 75 min of vigorous-intensity aerobic activity per week—is associated with major cardiovascular risk factors and is responsible for 5% of the global burden of acute myocardial infarction as well as 8% of all-cause mortality [53]. The harmful effect of sedentary time is independent of participation in physical activity [49]. A meta-analysis of data from more than one million individuals concluded that 1 h of physical activity eliminates the detrimental effects of 8 h of inactivity [54]. The high prevalence of physical inactivity (66% in European coronary patients [5]) leads to nearly as many disability-adjusted life years and deaths as smoking [1].

Concerns about the risk of adverse cardiovascular events [49] and kinesiophobia could potentially discourage coronary patients from physical activity. For healthy individuals, the risk of adverse cardiovascular events during physical activity is extremely low and by far outweighed by the benefits [50]. Sedentary subjects and those with cardiovascular risk factors should start physical activity at low intensity and progress gradually. One prospective study in 4846 coronary patients observed an almost negligible level of risk associated with high-intensity exercise (1 fatal cardiac arrest in 129,456 exercise hours), also compared to moderate-intensity exercise (1 non-fatal cardiac arrest in 23,182 h) [52]. Considering the low incidence of adverse events and the significant benefits, Rognmo et al. concluded that high-intensity exercise should at least be considered in coronary patients, ideally preceded by an exercise test [55].

Guidelines recommend that healthcare professionals should regularly assess and counsel on physical activity, promote engaging in physical activity and, if levels are insufficient, support efforts to increase physical activity levels [56]. Physical activity should be integrated into daily life, for example by choosing active modes of travelling (cycling or walking), taking breaks from sitting and reducing screen time, in order to minimise the amount of time spent being sedentary [56]. Internet- and phone-based programmes offer remote solutions to successfully increase physical activity in coronary patients [57]. Such interventions can include tailored coaching, guidance on goal setting and objectively measuring physical activity (and progress) with pedometers [57, 58].

In conclusion, emphasis on the harmful effects of sedentary time is as important as promoting exercise in patients with ASCVD. Promising research in the area of technology-based programmes suggests that personalised (digital) coaching may further increase participation and deliver individualised behavioural support to assist patients in remaining physically active in the long term.

## Stress management

Chronic mental stress was associated with a 2.1 times higher risk of myocardial infarction in the INTERHEART study [59]. Other studies show that chronic stress at work is associated with ASCVD events in men (RR ~ 1.2–1.5) [60] and long-term stressful conditions in family life increase ASCVD risk (RR ~ 2.7–4.0) [61, 62]. A Swedish population-based, sibling-controlled cohort study (*n* = 136,637) found an HR of 1.64 (95% CI 1.45–1.84) for any cardiovascular disease during the 1st year after the diagnosis of any stress-related disorder [63]. Furthermore, acute stress may trigger acute events, including arrhythmias and acute myocardial infarction [64]. These studies demonstrate a relationship between ASCVD and stress comparable to, or even stronger than, the relationship between elevated cholesterol or hypertension and ASCVD [65]. Advice on improvement of lifestyle risk factors should therefore include stress management [66, 67].

The biological processes associated with chronic stress and cardiovascular disease include high blood pressure, elevated lipids such as cholesterol, and inflammation-related molecules. These factors cause damage to the vessel wall and contribute to the gradual development of atherosclerotic plaques [65, 68]. Acute stressors lead to neurological and metabolic derangements that serve as possible triggers for an ACS [64, 67].

Practical tips to address ‘common’ mental stress (in response to challenges in everyday life) in patients include (1) talking about problems in the past tense, about solutions in the future tense; (2) focusing on health instead of pathology; and (3) focusing on solutions instead of challenges [69]. Cardiac rehabilitation provides psychosocial benefit to both men and women to a similar degree, but in different psychosocial indicators [70, 71]. Two randomised controlled trials (*n* = 362 and *n* = 237) showed that offering 20 sessions of stress management training during 1 year to patients after an ACS decreased the risk of recurrent ASCVD events (HR 0.59, 95% CI 0.42–0.83; *p* = 0.002) [72] and all-cause mortality in women (odds ratio 0.33, 95% CI 0.15–0.74; *p* = 0.007) [73]. In the case of clinically significant symptoms of depression, anxiety or hostility, referral for psychotherapy or collaborative care should be considered [3, 71, 74]. A randomised controlled trial (*n* = 235) showed that treatment for depression in older patients without ASCVD resulted in a significantly lower risk for developing first CAD events compared with usual care (28% vs 47%; HR 0.52, 95% CI 0.31–0.8) [75]. An internet-delivered cognitive behavioural therapy in depressed patients with high ASCVD risk (*n* = 562) significantly improved depressive symptoms and adherence to medication, and improved levels of physical activity [76].

In conclusion, mental stress affects the development of coronary disease, can trigger events, and may act as a barrier against beneficial behavioural changes. It remains to be proven if specific stress-modification strategies can decrease patients’ risk for ASCVD events. However, stress should be addressed by all healthcare professionals to support adherence and beneficial lifestyle behaviours [3, 66, 74]. Common stress in everyday life can be addressed through empathic, solution-focused communication [69], whereas clinically significant mental disorders mandate referral to other professionals [3, 71, 74].

## Implementation in practice

Implementation and long-term adherence are key in lifestyle management, but also constitute important challenges. Ideally, a multi-level, concerted effort by all involved parties, including policy makers, may contribute to an environment in which a healthy lifestyle to prevent ASCVD can be established and maintained. Healthcare professionals also have opportunities to facilitate successful implementation of and adherence to beneficial lifestyle change, e.g. by patient-centred communication [77] and as a role model by following healthy lifestyles themselves [78].

Patient-centred communication and shared decision-making between healthcare professionals and patients (including partner and family) form the foundation of motivation and commitment [79]. Cognitive-behavioural strategies (such as motivational interviewing) increase motivation and self-efficacy [80]. In addition, effective communication facilitates behavioural change by assessing the patients’ thoughts, attitudes, beliefs, and values regarding lifestyle change [69, 77], which helps to set realistic goals that fit in with life goals and are integrated in daily life [79]. In this regard, active partner participation in lifestyle modification results in better success rates [6, 81].

Lifestyle interventions, involving multidisciplinary healthcare professionals (e.g. nurses, dieticians, psychologists) and targeting multiple lifestyle aspects, enhance illness-coping strategies, improve treatment adherence, and reduce ASCVD events and mortality [82]. Nurse-coordinated programmes improve risk factor control [81], medication optimisation, and reduce non-cardiac emergency room presentations [6, 83].

A window of opportunity for lifestyle change occurs at the time of ASCVD diagnosis or (invasive) treatment. In the acute hospital admission setting, the importance of preventive measures can be emphasised directly and acutely. Appropriate prevention, including lifestyle changes, should be initiated before hospital discharge, as the probability of success tends to decrease rather than increase after discharge [8].

Once lifestyle change is initiated, long-term maintenance is challenging. Participation in a cardiac rehabilitation programme after hospitalisation is recommended for all cardiovascular patients [3]. Long-term support may be needed [79]: an intensive multifactorial intervention over 3 years was compared to usual care and resulted in sustained improvements in physical activity, diet, and total cholesterol throughout the study, as well as a significant decrease of fatal and non-fatal cardiovascular events [84].

Potentially, tailor-made lifestyle interventions that meet the needs for specific age, gender, cultural, socio-economic, comorbidity or risk-based groups could lead to greater success regarding lifestyle modification [79].

## Conclusion

Lifestyle modification continues to be a cornerstone of ASCVD prevention. Healthcare professionals have a responsibility in the clinical setting to address an unhealthy lifestyle and should know how to support a beneficial lifestyle. Even minimal efforts to stimulate healthy lifestyles in patients can have a significant impact, especially if targeted at individuals with a high estimated ASCVD risk and a high probability of success. Yet, achieving meaningful and persisting lifestyle changes is challenging, and better outcomes are expected when behavioural change is incorporated into daily life and in line with personal patient values. In individuals at very high cardiovascular risk, referral is recommended to programmes that offer multimodal behavioural interventions, integrating physical activity, nutrition, stress management, smoking cessation support, and counselling on psychological risk factors. Investment in the implementation of and adherence to a healthy lifestyle should receive the highest priority to continue to reduce the burden of ASCVD.

**Fig. 1:**
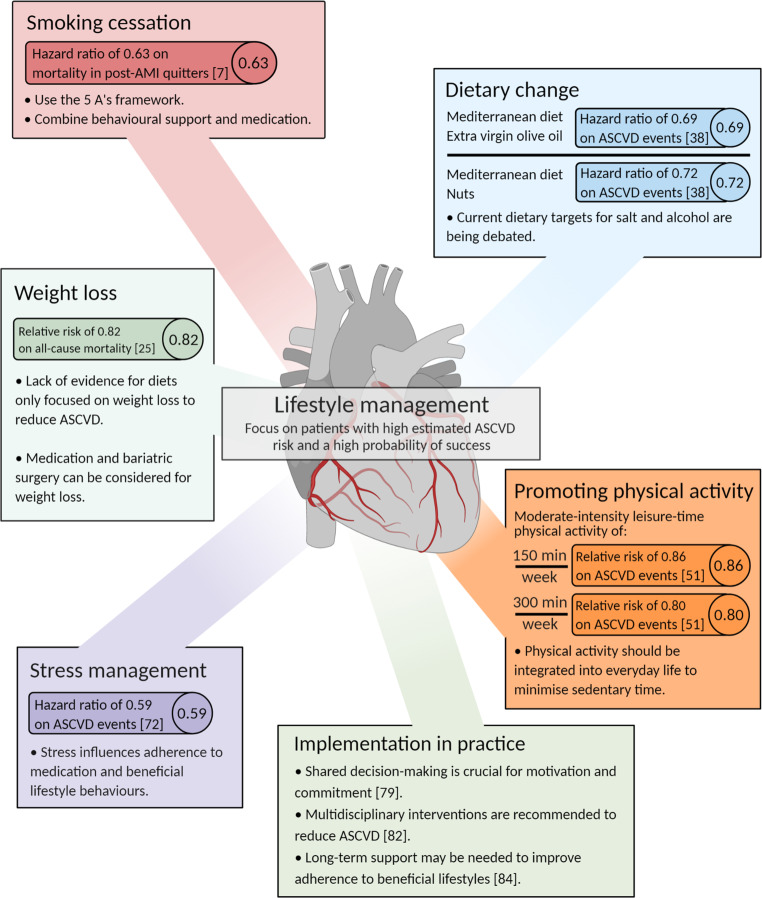
Key messages. *Post-AMI* post-acute myocardial infarction, *ASCVD* atherosclerotic cardiovascular disease
